# Adjusting effective multiplicity ($M_{\mathrm{eff}}$) for family-wise error rate in functional near-infrared spectroscopy data with a small sample size

**DOI:** 10.1117/1.NPh.11.3.035004

**Published:** 2024-07-27

**Authors:** Yuki Yamamoto, Wakana Kawai, Tatsuya Hayashi, Minako Uga, Yasushi Kyutoku, Ippeita Dan

**Affiliations:** a Chuo University, Applied Cognitive Neuroscience Laboratory, Faculty of Science and Engineering, Tokyo, Japan; b Yamato University, Department of Information Science, Faculty of Science and Engineering, Osaka, Japan; c Health Science University, Department of Welfare and Psychology, Faculty of Health Science, Yamanashi, Japan

**Keywords:** functional near-infrared spectroscopy, family-wise error, multiple comparisons, effective multiplicity (*M*_eff_), statistical analysis

## Abstract

**Significance:**

The advancement of multichannel functional near-infrared spectroscopy (fNIRS) has enabled measurements across a wide range of brain regions. This increase in multiplicity necessitates the control of family-wise errors in statistical hypothesis testing. To address this issue, the effective multiplicity ($M_{\text{eff}}$) method designed for channel-wise analysis, which considers the correlation between fNIRS channels, was developed. However, this method loses reliability when the sample size is smaller than the number of channels, leading to a rank deficiency in the eigenvalues of the correlation matrix and hindering the accuracy of $M_{\text{eff}}$ calculations.

**Aim:**

We aimed to reevaluate the effectiveness of the $M_{\text{eff}}$ method for fNIRS data with a small sample size.

**Approach:**

In experiment 1, we used resampling simulations to explore the relationship between sample size and $M_{\text{eff}}$ values. Based on these results, experiment 2 employed a typical exponential model to investigate whether valid $M_{\text{eff}}$ could be predicted from a small sample size.

**Results:**

Experiment 1 revealed that the $M_{\text{eff}}$ values were underestimated when the sample size was smaller than the number of channels. However, an exponential pattern was observed. Subsequently, in experiment 2, we found that valid $M_{\text{eff}}$ values can be derived from sample sizes of 30 to 40 in datasets with 44 and 52 channels using a typical exponential model.

**Conclusions:**

The findings from these two experiments indicate the potential for the effective application of $M_{\text{eff}}$ correction in fNIRS studies with sample sizes smaller than the number of channels.

## 1 Introduction

### 1.1 fNIRS

Functional near-infrared spectroscopy (fNIRS) is a noninvasive and convenient neuroimaging tool that has gained popularity over the last few decades.^1^^–^^4^ It measures cerebral hemoglobin concentration changes following neuronal activation by shining NIR light (650 to 950 nm) onto the head and detecting the reflecting light that propagates through biological tissue. Jöbsis^5^ reported the first noninvasive measurement of living tissue in humans using this technique. Several research groups reported that fNIRS was effective in capturing cerebral hemodynamic responses associated with brain activity.^6^^–^^9^ Since then, fNIRS has evolved as an established tool in functional neuroimaging and has been applied in various studies beyond the realm of conventional neuroimaging including early developing brains,^10^ universal everyday brain activities,^11^ interpersonal social interactions,^12^ and others.

fNIRS is compact, highly portable, and tolerant to body motion, allowing measurement of brain activity in everyday environments without disturbing the subject’s natural behavior, especially because of its wearability.^13^ Such flexibility makes fNIRS suitable for a broad range of experimental designs and age groups, extending its use beyond basic research in cognitive neuroscience to diverse fields.

### 1.2 Multiple Comparison Problem Due to Increased Number of Channels

Advancements in fNIRS technology have led to significant expansion in the number of channels and measurement points, necessitating multiple comparison corrections to effectively control type I errors. Originally, fNIRS measurement began with single-channel systems, comprising just one transmitter and one receiver. Later, due to light interference between adjacent channels, wide spacing on the scalp was necessary, limiting the number of channels to only a few. To overcome this limitation, Maki et al.^14^ introduced multichannel fNIRS that included a frequency encoding system, enabling simultaneous monitoring of multiple brain regions. Since then, with the progression of multichannelization, the number of channels used for standard fNIRS studies has gradually increased to several dozen. Also, whole-head measurements using more than 100 channels have been implemented.^15^ Furthermore, diffuse optical tomography (DOT), an advanced form of fNIRS, estimates the signal source by integrating both short- and long-distance channels and makes it possible to reconstruct a three-dimensional (3D) image of the functional hemodynamic response.^16^^–^^20^ With this ability to reconstruct continuous image data, a significantly large number of channels compared to the number of measurement points could be handled.

Advancements in the multichannelization of fNIRS has enabled measurements of a wide range of brain regions. However, also due to this advancement, addressing the issue of multiple comparisons in statistical hypothesis testing in fNIRS analysis has become necessary. In standard fNIRS analysis, statistical hypothesis tests, such as the t-test and the analysis of variance (ANOVA), are conducted based on summary statistics obtained from first-level analysis to determine whether the activation level in a particular cognitive state is significant. With multichannel fNIRS, the multiplicity is equal to the number of channels, as a null hypothesis is set for each channel. Thus, as the number of channels increases, so does the risk of type I errors (false positive), in which at least one correct null hypothesis among all hypotheses is rejected. In other words, there is a risk of erroneously treating one or more nonactivated channels as activated channels. Therefore, the risk of type I errors must be controlled as family-wise errors (FWEs) in multichannel fNIRS analyses.

### 1.3 Multiple Comparison Problem in fNIRS Analysis

fNIRS data are often represented as channel-wise data, where the multiplicity is equal to the number of channels. When conducting multiple comparisons across multiple channels, family-wise error rate (FWER) can be calculated using the significance level (α) and the number of channels (M), as shown in the following equation:

$$\alpha_{\mathrm{FWE}}=1-{\left(1-\alpha \right)}^{M}.$$ (1)

Here, assuming α=0.05 and M=52 (a typical setting for 52 channels in a single-factor fNIRS analysis), the risk of false positives increases, resulting in $\alpha_{\mathrm{FWE}}≒0.93$.

The most typical control for FWE is the Bonferroni correction, which adjusts the significance α to $\alpha_{\mathrm{Bonf}}$ by dividing it by M to achieve $\alpha_{\mathrm{FWE}}≒0.05$:

$$\alpha_{\mathrm{Bonf}}=\frac{\alpha }{M}.$$ (2)

In the example above, this results in $\alpha_{\mathrm{Bonf}}≒0.00096$ (M=52, $\alpha_{\mathrm{FWE}}≒0.0499$), effectively suppressing type I errors. However, as M increases, the Bonferroni method can be too stringent, thereby increasing the risk of type II errors or false negatives.

One alternative to the conservative Bonferroni correction is Holm’s correction, which utilizes a step-down approach to enhance increased statistical power.^21^^,^^22^ Another alternative is the false discovery rate (FDR) method that targets the proportion of false positives among all significant findings.^23^ The FDR-based procedure can yield more statistical power than the Bonferroni method and be more robust against variations in the number of channels within regions of interest (ROIs).^24^

Although these methods yield greater statistical power compared to the Bonferroni method, they all begin with the same multiplicity, which is equal to the number of channels, in order to control type I errors for the most active channels.^25^ In other words, Bonferroni correction, Holm’s correction, and FDR methods require at least one test to exhibit a probability of significance lower than α/M. It is crucial to recognize that in multichannel fNIRS data, channels are not completely independent due to the correlations between them. Treating each channel as independent can lead to an overestimation of FWEs. Therefore, applying these methods without consideration of channel correlation might result in overcorrection.

### 1.4 Effective Multiplicity ($M_{\text{eff}}$)

Uga et al.^25^ demonstrated that effective multiplicity ($M_{\text{eff}}$) can be an effective approach to fNIRS analysis, accounting for correlations between channels. The $M_{\text{eff}}$ correction method was originally developed by Cheverud^26^ for multiple-testing corrections in genetic studies. In the $M_{\text{eff}}$ approach, eigenvalue decomposition is applied to a correlation matrix derived from a dataset with inherent correlations. This process yields eigenvalues that reflect the magnitude of correlations between each data point. These eigenvalues are used to estimate $M_{\text{eff}}$, which represents the number of independent tests. Consequently, α is corrected by $M_{\text{eff}}$ instead of M:

$$\alpha_{M_{\mathrm{eff}}}=\frac{\alpha }{M_{\mathrm{eff}}}.$$ (3)

Here we will describe the theoretical framework of the $M_{\text{eff}}$ method for a typical fNIRS data structure. It is crucial to recognize that the $M_{\text{eff}}$ method, which was originally invented for genetic data, has been modified to fit the fNIRS data structure. For multichannel fNIRS data obtained from M channels across N subjects, summary data for group analysis is represented as $\beta^{M\times N}$. Then from the $\beta^{M\times N}$ correlation matrix (M×M), the eigenvalue vector ($\lambda_{i}$) is derived as follows:

$$\lambda_{1},\lambda_{2},\ldots \lambda_{M}.$$ (4)

Previous studies have shown that the total correlation among a dataset can be quantified by the variance of the eigenvalues ($V_{\lambda }$) derived from a correlation matrix. Utilizing this property, Cheverud^26^ proposed estimating $M_{\text{eff}}$ as

$$M_{\mathrm{eff}}=1+(M-1)(1-\frac{V_{\lambda }}{M}),\;V_{\lambda }=\overset{M}{\underset{i=1}{\sum }}\frac{{\left(\lambda_{i}-1\right)}^{2}}{M-1}.$$ (5)

This equation accounts for two extreme situations. When tests are completely independent, each eigenvalue equals 1, resulting in the equation $M_{\text{eff}}=M$. Conversely, when tests are completely identical, the primary eigenvalue is M, and all subsequent eigenvalues are 0, leading to $M_{\text{eff}}=1$. Although Cheverud’s^26^ equation accurately estimates $M_{\text{eff}}$ at these two extremes, it tends to overestimate $M_{\text{eff}}$ in intermediate situations, leading to excessively conservative results.

Following a modification by Li and Ji,^27^ however, Galwey^28^ proposed a generalized function that overcomes this objection:

$$M_{\mathrm{eff}}={\left(\overset{M}{\underset{i=1}{\sum }}\sqrt{\lambda_{i}}\right)}^{2}/\overset{M}{\underset{i=1}{\sum }}\lambda_{i}.$$ (6)

Uga et al.^25^ adopted Galwey’s function because this method was optimized for multiple signals with strong correlations and could be applied continuously. Applying the $M_{\text{eff}}$ correction to three kinds of experimental data with different activation patterns and performing resampling simulations resulted in the $M_{\text{eff}}$ values being 10 to 15 out of 44 channels of data.^25^ This indicates that the $M_{\text{eff}}$ approach provides an effective correction for multichannel fNIRS data.

### 1.5 Rank Deficiency Problem in $M_{\text{eff}}$

Although the $M_{\text{eff}}$ approach is beneficial for channel-wise fNIRS statistical analysis, there is a nonnegligible concern about the impact of sample size (N) on its reliability. Typically, fNIRS studies involve relatively small N. This often results in situations where N is smaller than M. Specifically, when N is smaller than the number of variables (i.e., N<M), the correlation matrix can have a maximum of N−1 nonzero eigenvalues, with the remaining eigenvalues being zero. Therefore, in the context of fNIRS statistical analysis, applying the $M_{\text{eff}}$ method to data where N is smaller than M can lead to an underestimation of $M_{\text{eff}}$ due to the rank deficiency of the eigenvalues. When α is corrected for an underestimated $M_{\text{eff}}$, the risk of type II errors increases as a result of the less stringent correction. This issue emphasizes the need for a reevaluation of the application of $M_{\text{eff}}$ correction to multichannel fNIRS analysis.

### 1.6 Objective

Inspired by the challenges of rank deficiency, our study focused on evaluating the effectiveness of the $M_{\text{eff}}$ method in multichannel fNIRS data with a small N. We prepared four different sets of experimental data, each with different activation profiles, and conducted a two-step verification process comprising experiment 1 and experiment 2. For all datasets, N was greater than M, which enabled us to evaluate the validity of $M_{\text{eff}}$ for a wide range of N/M ratios. In experiment 1, we explored the relationship between N and $M_{\text{eff}}$ through random resampling simulations. In experiment 2, we applied a model to this relationship and performed simulations to examine the feasibility of estimating valid $M_{\text{eff}}$ from a small N. Based on these results, we discuss whether the $M_{\text{eff}}$ approach can be applied for FWE correction in fNIRS data with a small N.

## 2 Experiment 1

### 2.1 Methods

#### 2.1.1 Experimental data

In this study, we used four sets of experimental data with different activation profiles and N exceeding M (Table 1). Each dataset was obtained from our previous fNIRS experiments and they collectively provide data for a variety of participants performing a variety of cognitive tasks. They were also used in experiment 2. Below, we will describe the respective experimental procedures and participants. All participants, or their guardians in cases where participants were minors, provided informed consent, and each experiment was approved by the ethics committees of Chuo University (all tasks) and/or Jichi Medical University Hospital and the International University of Health and Welfare (placebo) and complied with the latest version of the Declaration of Helsinki.

**Table 1:** Summary of experimental data.

Experimental data	N	M	Summary data	Participant profile
Go/No-go task	66	44	Average values of oxy-Hb signal	Typically developing children
Verification of the placebo effect	116	44	Average values of oxy-Hb signal	Children with ADHD
Word translation task	88	52	β-values	Healthy adults
Stroop task	59	52	β-values	Healthy adults

#### 2.1.2 Go/No-go task

The participant sample for the Go/No-go task was 66 right-handed, typically developing children (38 boys and 28 girls, average age=8.3±1.8, age range 6 to 14 years). Inhibition-related cortical activation was measured during a Go/No-go task. In this study, fNIRS measurements were conducted using 44 channels. The experimental design, preprocessing, and calculation of summary data were consistent with previous studies.^29^^–^^31^ The procedure consisted of 6 block sets, containing alternating go (baseline) and Go/No-go (target) blocks, each block lasting 24 s. In the go block, participants were presented with a random sequence of two pictures and were asked to press a button for both pictures. In the Go/No-go block, participants were presented with a no-go picture, 50% of the time, requiring them to respond to half of the trials (go trials) and inhibit their response to the other half (no-go trials). From the preprocessed time series data, channel-wise and participant-wise contrasts were computed as the summary data by calculating the intertrial mean of differences between the oxygenated hemoglobin (oxy-Hb) signals for target periods (4 to 24 s after the Go/No-go block onset) and baseline periods (14 to 24 s after the go-block onset).

#### 2.1.3 Verification of placebo effect

Participants for the verification of placebo effects sample were 116 right-handed children with attention deficit hyperactivity disorder (ADHD) (92 boys and 24 girls, average age=8.1±1.9, range 6 to 14). Data were extracted from published studies,^29^^,^^30^^,^^32^^,^^33^ and a detailed description will be published elsewhere (in preparation). Data obtained from randomized, double-blind, crossover, placebo-controlled design trials using methylphenidate (MPH), or atomoxetine (ATX) were analyzed. Participants were examined twice, with an interval of at least 4 days but within 30 days. On each examination day, participants completed two sessions: one before medication (active drug or placebo) administration and the other at 90 min after medication. Those who were administered an active drug on the first day were administered a placebo on the second day, whereas those who were administered a placebo on the first day were administered an active drug on the second day. Placebo effects were assessed by examining brain activation during the Go/No-go task. In this study, fNIRS measurements were conducted using 44 channels. The experimental design, preprocessing, and calculation of summary data were consistent with those described above. To assess the placebo effect, the intraplacebo contrast, which is the difference between post- and preadministration contrasts for placebo participants, was calculated.

#### 2.1.4 Word translation task

Participants that did the word translation task were 88 healthy right-handed Japanese young adults (15 participants were excluded; 42 males, and 46 females, average average age=20.0±1.4, age range 18 to 23 years). In this study (submitted), fNIRS measurements were conducted using 52 channels. The experimental design, preprocessing, and calculation of summary data were consistent with a previous study.^34^ The stimuli were divided into nontranslation baseline blocks and task blocks. There were four task conditions in the task blocks: translation direction (English-into-Japanese/Japanese-into-English)×familiarity(high/low familiarity). In Japanese-into-English task blocks, participants were asked to translate Japanese words written in red into the corresponding English words and to type them. In the English-into-Japanese task blocks, they were asked to translate English words written in red in the Roman alphabet into corresponding Japanese words and to type their translation in the Roman alphabet. Individual timeline data for the oxy-Hb signal of each channel were preprocessed. General linear model (GLM) analysis^35^ was conducted, and β-values, indicating the degree of activation, for each individual on each channel were used as summary data. For the present experiment, we chose to use data from the “English-into-Japanese/High Familiarity” condition for our analysis because the activation patterns were most similar between groups.

#### 2.1.5 Stroop task

Participants for the Stroop task were 59 healthy, right-handed, Japanese young adults (2 participants were excluded; 30 males and 29 females, average age=21.8±0.96, age range 20 to 24 years). In this study (in preparation), fNIRS measurements were conducted using 52 channels. Participants were presented with a word stimulus indicating a color that was printed in the same or in a different color. The task had three conditions: congruent, incongruent, and neutral. In the congruent condition, the ink color was consistent with the meaning of the word (e.g., “red” written in red). In the incongruent condition, the ink color was not consistent with the meaning of the word (e.g., “green” written in red). In the neutral condition, participants were only required to name the ink color (e.g., “XXX” written in red) without judging the meaning of a word. There are two types of Stroop tasks in neuroimaging experiments: identifying a color name (task I) and judging the correspondence of an ink color and the meaning of a word (task II). Participants were divided into two groups: one group first engaged in task I and then in task II, whereas the other group started with task II, followed by task I. For task II, a different brain activity was observed between the two groups, suggesting the occurrence of a sequential effect. Consequently, only task 1, where no order effect was observed, was utilized for analysis in this study. For the first level analysis, the individual timeline data for oxy-Hb signal were analyzed. Channels with a signal variation of 10% or less due to defective measurements were excluded from the analysis. After the exclusion, wavelet minimum description length (Wavelet-MDL) was applied to remove the effect of measurement noise, such as breathing and cardiac movement from the remaining channels.^36^ GLM analysis^35^ was conducted and the β-values for each individual on each channel were used as summary data. Specifically, the contrast between the incongruent and the neutral conditions was calculated as Stroop interference where a larger contrast indicates greater cognitive interference.

#### 2.1.6 Resampling simulation

We reanalyzed the four kinds of experimental data described above. The β-values of a GLM or average values of oxy-Hb signals were used as summary data. To elucidate the relationship between N and $M_{\text{eff}}$, we randomly resampled N from the minimum to the maximum and calculated the $M_{\text{eff}}$ for each dataset. For each N, resampling was performed 1000 times, and the average value and standard deviation (SD) were calculated. To calculate $M_{\text{eff}}$, we utilized Galwey’s function, as was done in an earlier study.^25^ The M was fixed based on the actual number used for measurements (44 or 52 channels). We set the minimum N for simulations at 3, due to the requirement of having at least three data points to compute SD, which is essential in calculating the correlation coefficient. These simulations were conducted using MATLAB R2023a (MathWorks, Inc., Natick, Massachusetts, United States).

### 2.2 Results

**Fig. 1:**
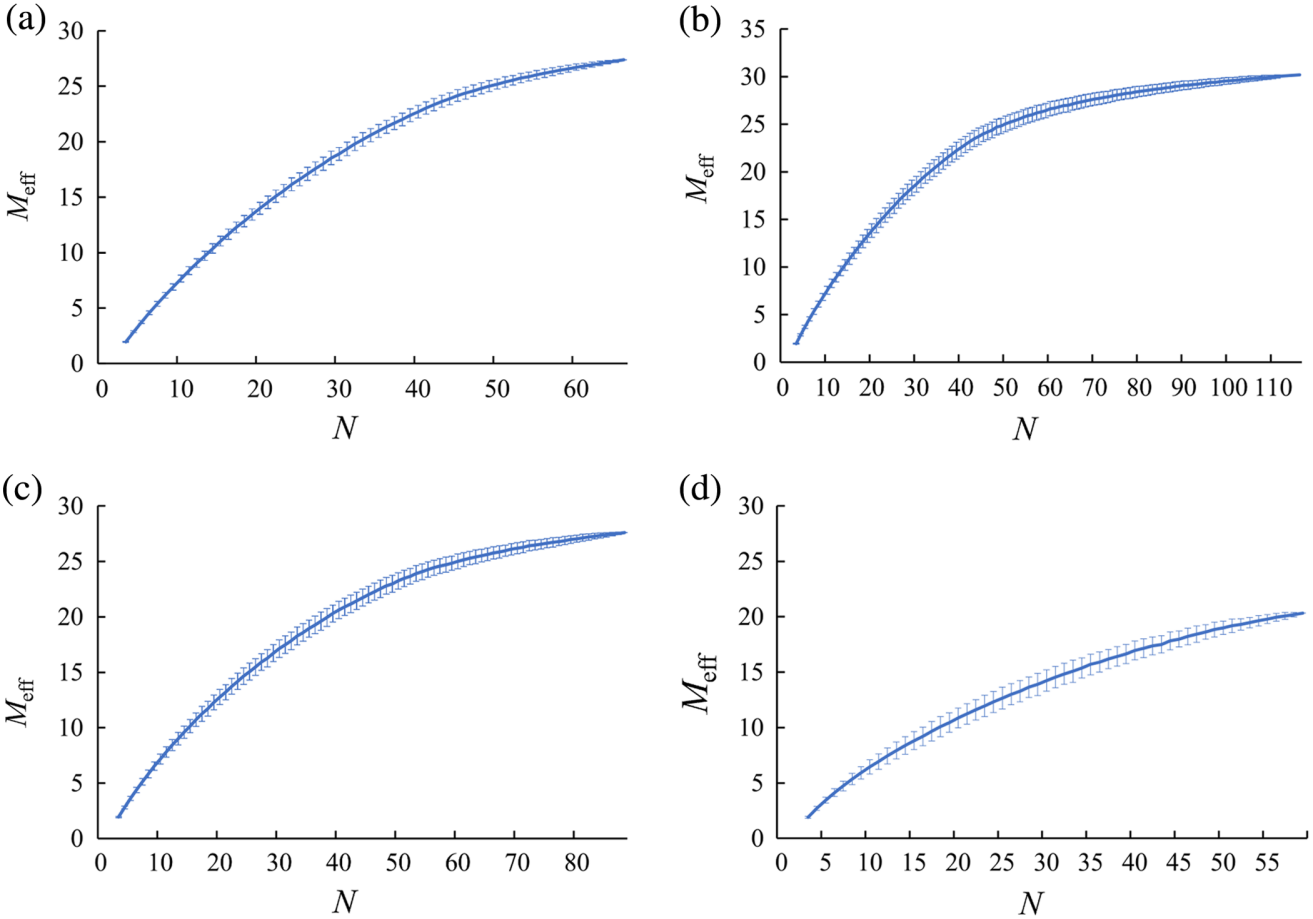
Average $M_{\text{eff}}$ values for the total number of N for each type of experimental data: (a) Go/No-go task (M=44), (b) verification of the placebo effect (M=44), (c) word translation task (M=52), and (d) Stroop task (M=52). Error bars indicate SD.

We plotted the average $M_{\text{eff}}$ values along with the SD for each N for each dataset (Fig. 1). For each dataset, $M_{\text{eff}}$ values displayed a monotonic exponential increase when N was smaller than M. On the other hand, as N surpassed M, the rate of increase gradually decreased and finally converged to a constant value. Specifically, in the datasets derived from the Go/No-go task and the verification of the placebo effect (M=44), the $M_{\text{eff}}$ values exhibited a monotonic increase up to approximately N=44. Beyond this point, the rate of increase began to slow down. For the Stroop and word translation tasks (M=52), similar results were observed at around N=52.

### 2.3 Discussion

Random resampling simulations in this study revealed a consistent pattern of $M_{\text{eff}}$ values. We observed that when N is smaller than M, $M_{\text{eff}}$ values tend to increase exponentially and monotonically. However, once N exceeds a certain threshold, these increases hit a ceiling, and $M_{\text{eff}}$ values begin to converge to a constant value, showing only slight fluctuations even as N continues to increase. Thus when N is smaller than M, $M_{\text{eff}}$ is likely to be underestimated due to rank deficiency in calculating eigenvalues, potentially leading to less stringent FWE corrections. On the other hand, when N surpasses M, valid $M_{\text{eff}}$ can be obtained as all eigenvalues are included in the calculation.

This pattern, characterized by an initial sharp increase in the dependent variable followed by a convergence at a particular level, corresponds to the behavior of a typical exponential growth model. Based on such a model, it may be feasible to estimate $M_{\text{eff}}$ values even when N is smaller than M. In experiment 2, we aimed to estimate valid $M_{\text{eff}}$ values from a small N by modeling this observed relationship.

## 3 Experiment 2

### 3.1 Methods

#### 3.1.1 Making predictions using a typical exponential model

To predict a valid $M_{\text{eff}}$ from a small N, the plots obtained in experiment 1 were modeled. In this experiment, we assumed the following typical exponential model to describe the relationship between N and $M_{\text{eff}}$:

$$y=-ae^{-bx}+c.$$ (7)

This model demonstrates that as x increases, y grows exponentially, and eventually hits a ceiling. In this model, each parameter, a, b, and c, has specific roles: a controls the magnitude of growth, b determines the growth rate, and c sets the upper limit that y approaches as x increases.

Our objective was to estimate a valid $M_{\text{eff}}$ by fitting this model to the results of experiment 1 and identifying the parameters a, b, and c. The $M_{\text{eff}}$ values at N=M+1, where all eigenvalues were obtained and the rate of increase in $M_{\text{eff}}$ values began to decrease in experiment 1, were considered practical upper limits and set as the target values for prediction.

#### 3.1.2 Assessing model validity

The exponential model, used to explain the relationship between N and $M_{\text{eff}}$ proposed earlier, was evaluated for its validity. The model was fitted to the results of experiment 1 (3≤N≤M+1) using the nonlinear least square method. We assessed the model’s goodness of fit through the root-mean-squared error (RMSE). The index indicates the extent to which predicted values from the model deviate from the actual observed values. A lower RMSE, closer to 0, signifies a more accurate model.

In addition, when employing an exponential model, a logarithmic transformation can be applied to facilitate the handling of nonlinearity within a linear model framework. If a phenomenon follows the proposed model, its relationship can be transformed into a linear form through the following logarithmic transformations:

$$y=-ae^{-bx}+c\;c-y=ae^{-bx}\;\ln(c-y)=-bx+\ln\;a,$$ (8)

where a, b, and c stand for constants, and y and x correspond to $M_{\text{eff}}$ and N, respectively. If a plot of N against $\ln(c-M_{\mathrm{eff}})$ demonstrates a linear relationship, it is indicative of N and $M_{\text{eff}}$ conforming to an exponential. Based on this relationship, we examined N against $\ln(c-M_{\mathrm{eff}})$, using parameter c derived from fitting the exponential model to the results of experiment 1. The linearity of this relationship was evaluated by fitting a linear model, y=px+q, where p and q stand for constants, and y and x correspond to $M_{\text{eff}}$ and N, respectively. Model fitting was conducted using Python’s “curve fit” and “polyfit” functions from the “scipy.optimize” module and “NumPy” library, respectively. Furthermore, the RMSE was calculated using the “mean_squared_error” function from the “sklearn.metrics” library.

#### 3.1.3 Random resampling and predictive simulations

**Fig. 2:**
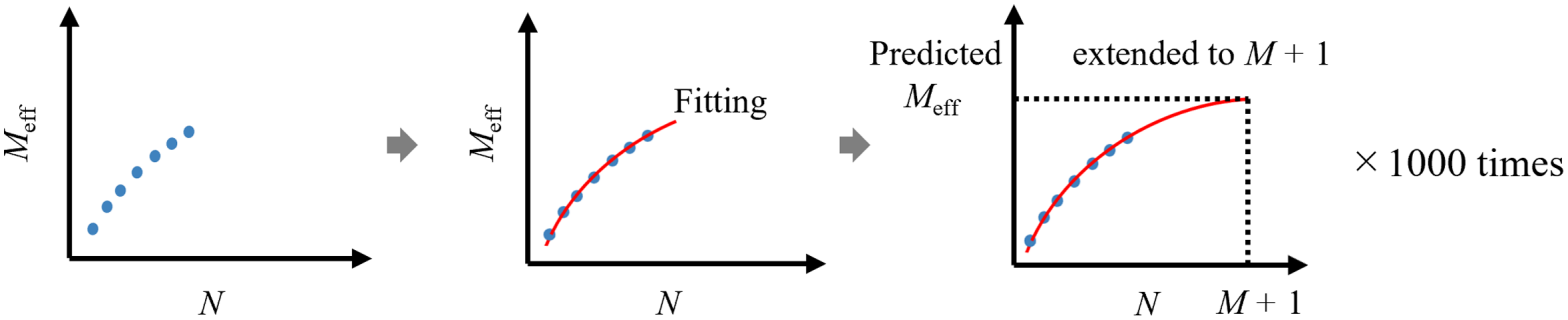
Resampling and predictive simulation.

Using simulations, we tested whether the valid $M_{\text{eff}}$ could be predicted from fNIRS datasets when N is smaller than M (Fig. 2). For each dataset, we performed random resampling from N=3 and up (3≤N≤M+1), and the $M_{\text{eff}}$ values for each N were calculated. Subsequently, an exponential model was fitted to the obtained $M_{\text{eff}}$ values to predict the $M_{\text{eff}}$ values at N=M+1. This process was replicated 1000 times for each N (3≤N≤M+1). The average and SD of the predicted $M_{\text{eff}}$ values for each N were plotted for comparison with target values. In addition, we computed the average and SD of the difference between the target and predicted values to represent prediction errors. The ratio of prediction errors to the target value was examined. Similar to the above, MATLAB R2023a (MathWorks, Inc., Natick, Massachusetts, United States) was used for the simulation.

### 3.2 Results

#### 3.2.1 Assessing model validity

**Fig. 3:**
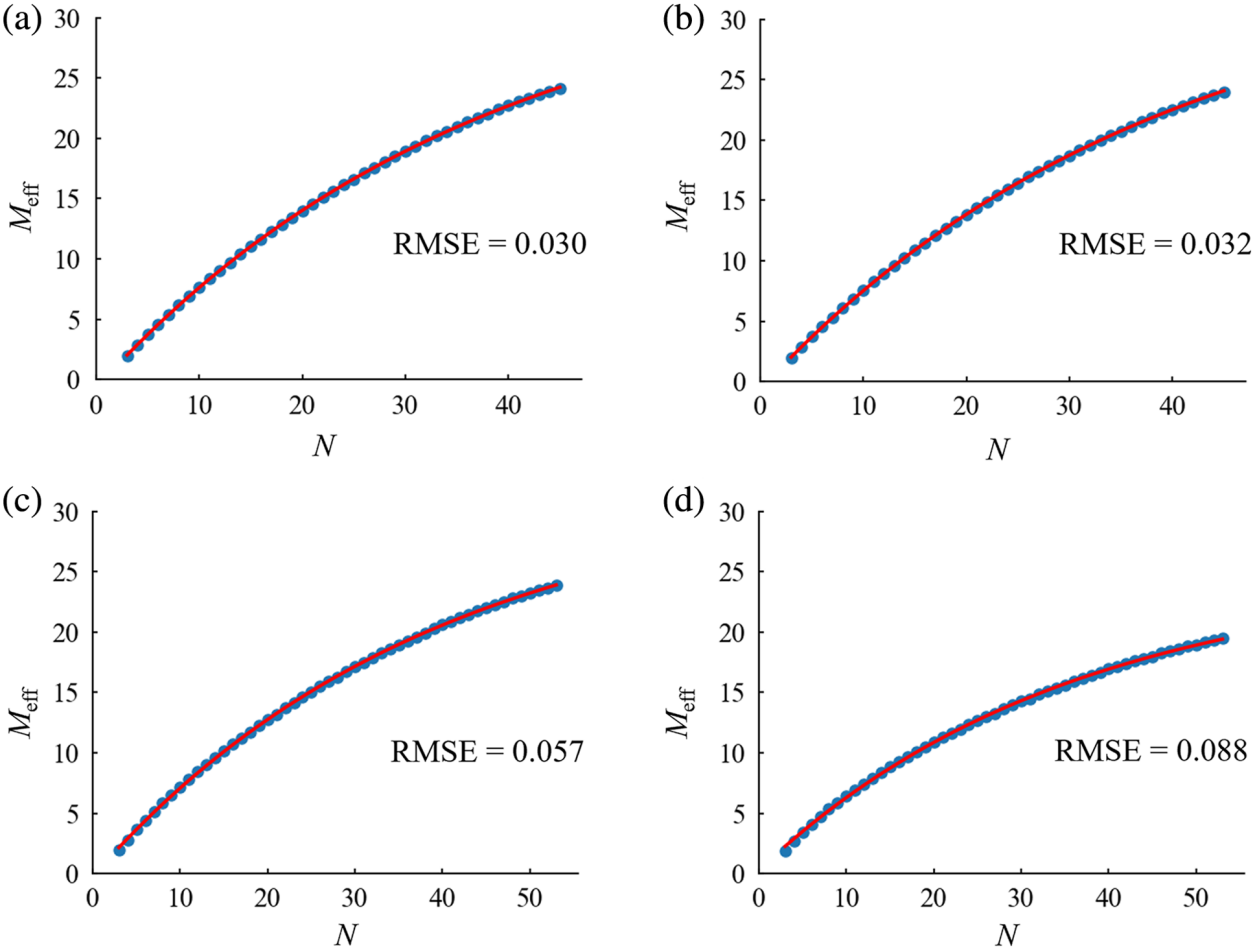
Exponential model fitting for $M_{\text{eff}}$ for each type of experimental data: (a) Go/No-go task (M=44), (b) verification of the placebo effect (M=44), (c) word translation task (M=52), and (d) Stroop task (M=52). The blue dots represent the $M_{\text{eff}}$ for each N (3≤N≤M+1). The red line indicates the curve resulting from the regression of the exponential model on $M_{\text{eff}}$ for each N.

**Fig. 4:**
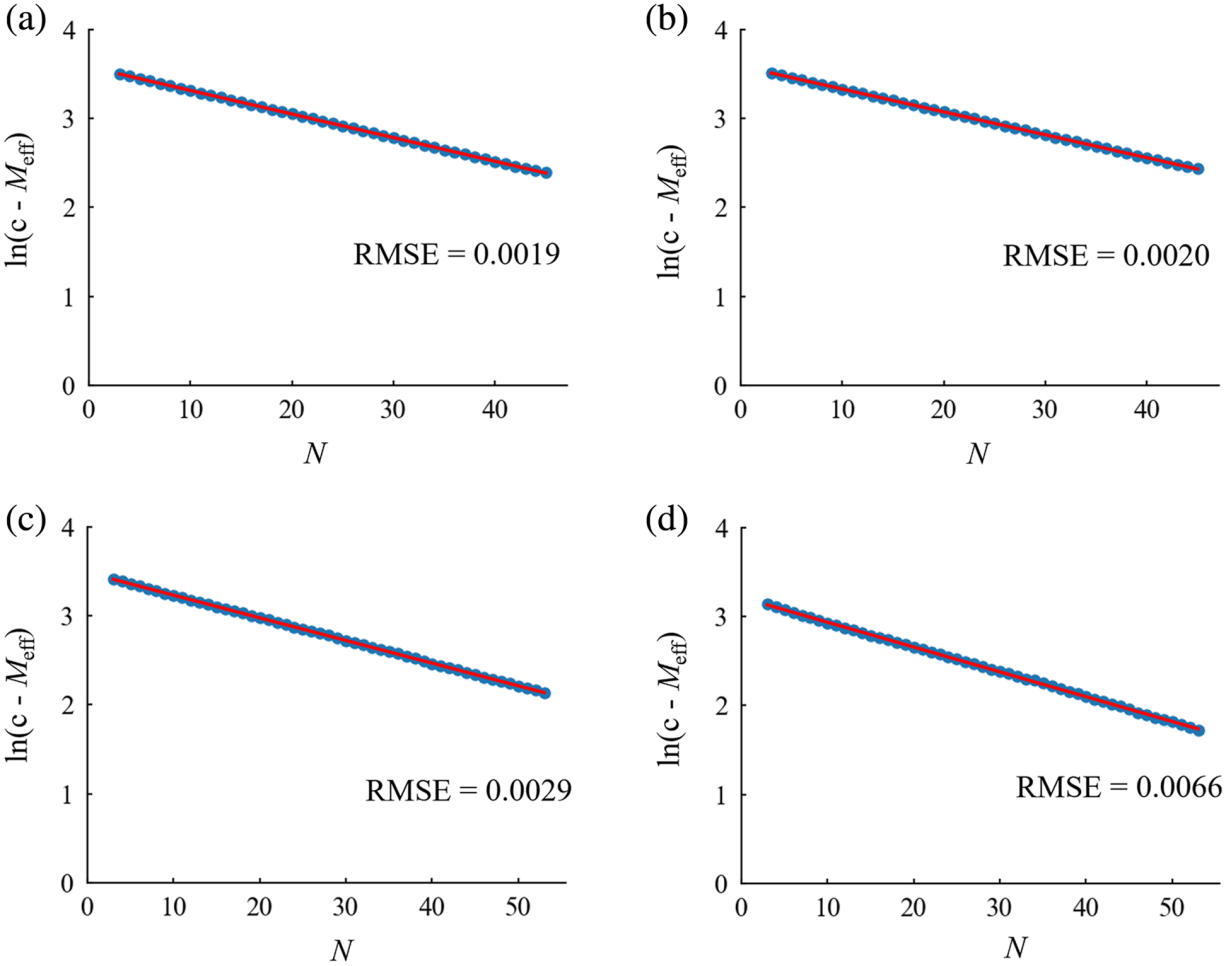
Linear model fitting for $\ln(c-M_{\text{eff}})$ for each type of experimental data: (a) Go/No-go task (M=44), (b) verification of the placebo effect (M=44), (c) word translation task (M=52), and (d) Stroop task (M=52). The blue dots show the values of $\ln(c-M_{\text{eff}})$ for each N (3≤N≤M+1). The red line represents the straight line obtained by regressing a linear model on $\ln(c-M_{\text{eff}})$ for each N.

For each dataset, the exponential model was fitted to the graph from experiment 1 in the range 3≤N≤M+1, and the goodness of fit was calculated (Fig. 3). Within each dataset, the RMSE was found to be notably small: <0.1. Subsequently, utilizing the parameter c obtained from this fitting, the relationship between N and $\ln(c-M_{\mathrm{eff}})$ was plotted, followed by the fitting of a linear model to this graph (Fig. 4). The RMSE for this linear model was also found to be low: <0.01.

#### 3.2.2 Resampling and predictive modeling simulations

**Fig. 5:**
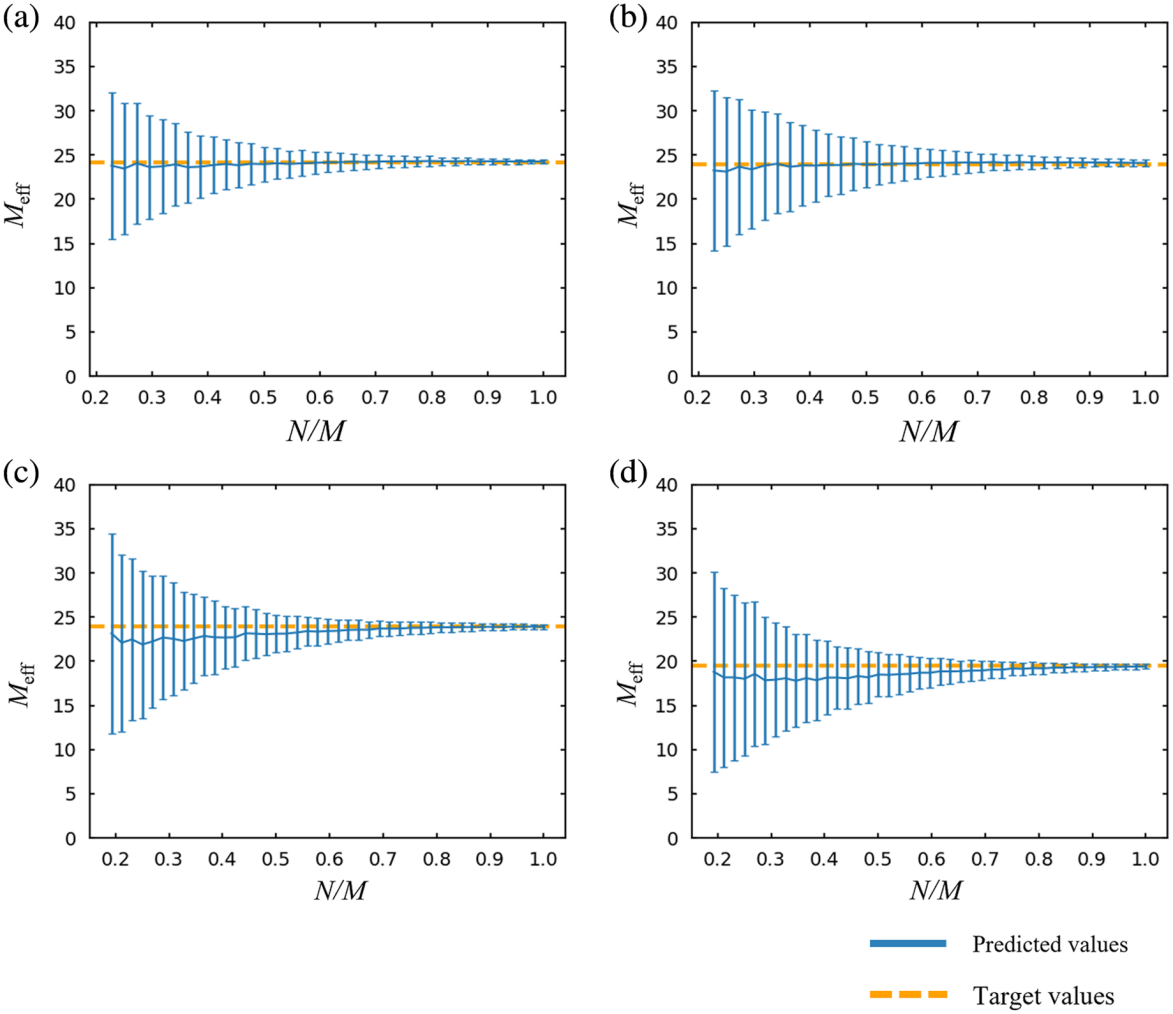
Comparison of predicted values and target values for each type of experimental data: (a) Go/No-go task (M=44), (b) verification of the placebo effect (M=44), (c) word translation task (M=52), and (d) Stroop task (M=52). The blue lines represent average predicted $M_{\text{eff}}$ values for each N/M. Error bars indicate SD. The orange lines indicate the true values of $M_{\text{eff}}$ of at N=M+1.

**Fig. 6:**
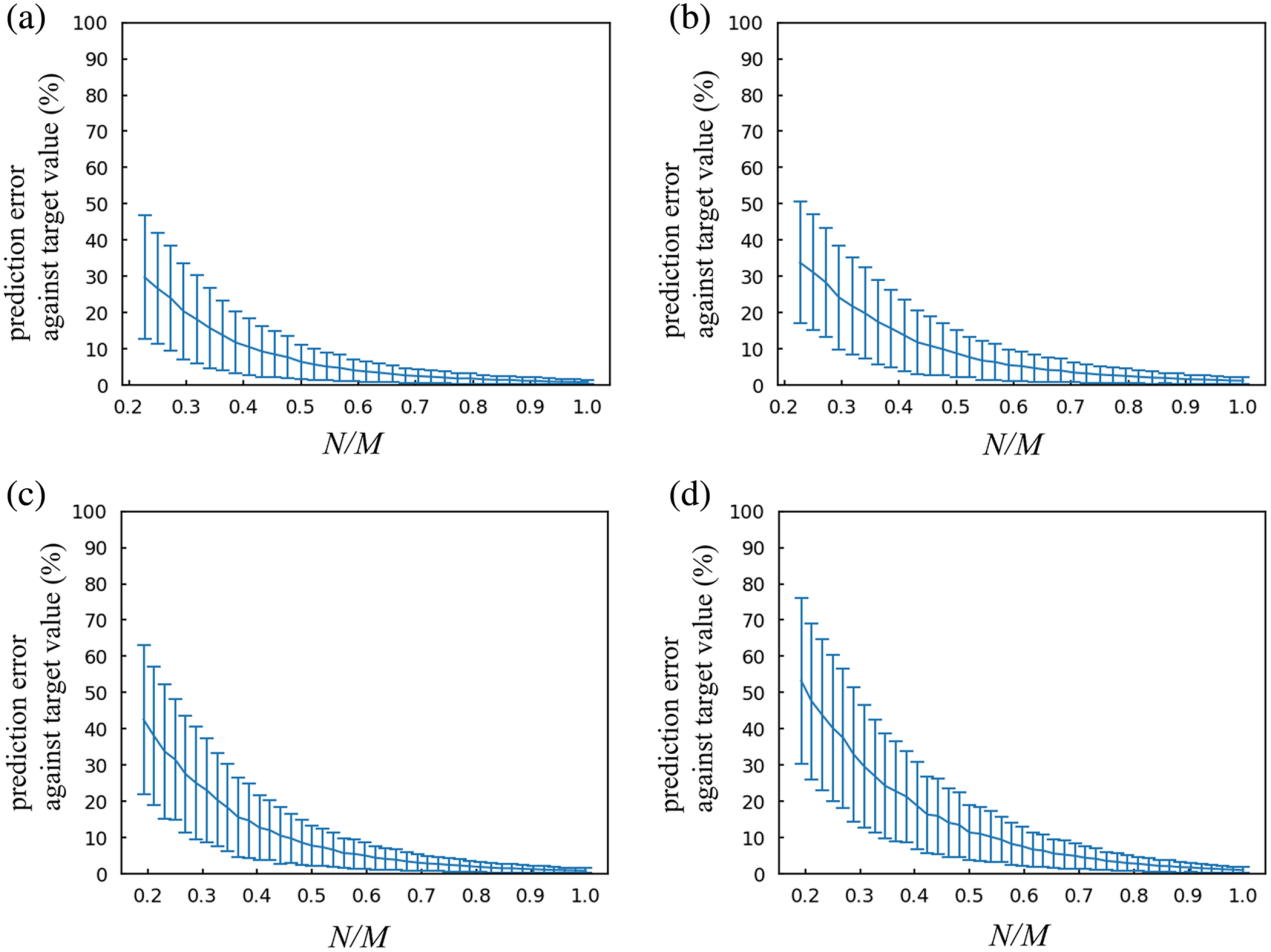
Average and SD of percentage of the prediction error against target values for each type of experimental data: (a) Go/No-go task (M=44), (b) verification of the placebo effect (M=44), (c) word translation task (M=52), and (d) Stroop task (M=52). Error bars indicate SD.

We graphically represented both the average and SD of the predicted $M_{\text{eff}}$ values for each N used in the prediction, along with the $M_{\text{eff}}$ values at N=M+1 in experiment 1 (Fig. 5). The average of the difference between predicted and target values was also calculated. The percentage of prediction error against the target values is indicated for each N/M (Fig. 6). As N increased, the average of the predicted values tended to converge toward the target value, and the SD decreased (Fig. 5). For the Go/No-go task data, the percentage decreased to less than 5% at N/M=0.57 (N=25). Similarly, for the verification of the placebo data, this percentage decreased to less than 5% at N/M=0.64 (N=28). For the word translation task data, these percentages decreased to less than 5% at N/M=0.62 (N=32). For the Stroop task data, this percentage decreased to less than 5% at N/M=0.69 (N=36).

### 3.3 Discussion

The relationship between N and $M_{\text{eff}}$ identified in experiment 1 was approximated with a typical exponential model. In this model, the $M_{\text{eff}}$ at N=M+1 was treated as the upper limit of the increasing N, where all eigenvalues were calculated and valid $M_{\text{eff}}$ were obtained. The small RMSEs indicate high goodness of fit, and the relationship between N and $M_{\text{eff}}$ is well explained by the exponential model. Simulation results using 44 or 52 channel datasets revealed that the average of predicted values converged to the target value of $M_{\text{eff}}$ when N ranged from 30 to 40, accompanied by a corresponding decrease in SD. This implies that for multichannel fNIRS data, a 60% to 70% N to M ratio is sufficient to correct for α using reasonable $M_{\text{eff}}$ values.

In this experiment, numerous predicted values were generated by repeatedly resampling from the datasets with a large N and then conducting curve fitting based on the plotted relationship between N and $M_{\text{eff}}$. In actual analysis, predicted values of $M_{\text{eff}}$ can be derived by fitting the model to curves from a resampling simulation similar to those in experiment 1.

## 4 General Discussion

### 4.1 Overview

In this study, we investigated the effectiveness of the $M_{\text{eff}}$ method for fNIRS data with small N. Experiment 1 exploited resampling simulations using several sets of experimental data with different neural activation profiles to examine the relationship between N and $M_{\text{eff}}$. We found that the $M_{\text{eff}}$ values monotonically increase when N is smaller than M. Conversely, $M_{\text{eff}}$ values tend to converge when N exceeds M. In datasets with a small N, the impact of rank deficiency in eigenvalues can lead to calculations that underestimate $M_{\text{eff}}$ values, posing a risk of insufficient correction. However, in datasets where N exceeds M, all eigenvalues are obtained, allowing for appropriate correction. Experiment 2 attempted to estimate valid $M_{\text{eff}}$ by assuming a typical exponential model based on the relationships revealed in experiment 1. The application of the model to the graph produced in experiment 1 resulted in RMSEs of <0.01. The small RMSEs indicated that the model successfully explained the relationship between N and $M_{\text{eff}}$. The simulation involving resampling and prediction showed that even when N is smaller than M, the predicted values are distributed near the target values. Using respective datasets with 44 and 52 channels, the applicability of the $M_{\text{eff}}$ correction was demonstrated for N of 60 to 70% relative to M. These results suggest that appropriate $M_{\text{eff}}$ correction can be achieved using the typical exponential model even when N is smaller than M.

### 4.2 Reevaluation of $M_{\text{eff}}$

The random resampling simulations conducted in experiment 1 indicate potential risks of false positives in previous studies with small N, suggesting a need for a more stringent application of the $M_{\text{eff}}$ correction method. However, $M_{\text{eff}}$ correction maintains a balance between type I and type II errors compared to the Bonferroni correction that increases in multiplicity with increasing M. In datasets with 44 and 52 channels, the $M_{\text{eff}}$ values typically ranged between 20 and 30 when there was a sufficiently large N. This implies that the $M_{\text{eff}}$ correction preserves power, probably due to interchannel correlations. These patterns were observed in all datasets, supporting that the $M_{\text{eff}}$ approach is a robust correction method regardless of the experimental task, as described in a previous study.^25^

In this study, we aimed to predict $M_{\text{eff}}$ in situations, where N exceeds M, using datasets with 44 and 52 fNIRS channels. Given that typical channel-wise measurements involve 40 to 50 channels, our approach is applicable to current practices. However, since prediction errors are inevitable, it is recommended to ensure a greater N than M when possible. However, even in cases where N is smaller than M, defining an ROI based on previous studies or pilot experiments can ensure the effectiveness of $M_{\text{eff}}$ correction. In confirmatory studies with predefined ROIs, the correction for FWE may not be a serious issue due to fewer hypotheses. Conversely, exploratory studies, which require a broader definition of ROI for identifying active channels, might benefit more from our findings, especially in estimating the optimal $M_{\text{eff}}$ for datasets where N is smaller than M.

### 4.3 Limitations and Future Prospects

In this study, which used datasets with 44 and 52 channels, the exponential model demonstrated the feasibility of applying $M_{\text{eff}}$ correction even for N/M ranging from 60% to 70% (N ranging from 30 to 40 participants). The use of 40 to 50 channel settings is common in current channel-wise measurements, suggesting the applicability of our approach. In recent years, however, fNIRS measurements utilizing around 100 channels have been conducted. In these cases, it is anticipated that a larger N than 30 to 40 is required for the simulations described in the current study. In scenarios where the percentage of N to M is notably low, further adjustment is required to prevent underestimation (see Supplementary Material). The current study primarily focused on channel-wise analysis. However, the DOT approach, which reconstructs continuous imaging data, is becoming a mainstream method in fNIRS studies. With DOT data, over 1000 channels are typically defined, far exceeding the number of conventional fNIRS. Moreover, these channels generate a continuous reconstructed image with thousands to million voxels^16^^–^^20^ for which distinct statistical considerations are necessary.^37^ Hence, the furthering of multichannelization imposes a limitation of the exponential model used in this study, suggesting that alternative approaches may be required for future validation.

Moreover, the application of $M_{\text{eff}}$ correction explored in this study, as in a previous study,^25^ is for one-sample t-tests. This is frequently used to test for significant activation in each channel. This approach is also applicable in paired designs where brain activities under different conditions are compared by taking the differences and applying one-sample t-tests. However, in fNIRS studies, two-sample t-tests or ANOVAs may sometimes be more suitable. In unpaired designs, it is impossible to consider differences of summary data. This makes it difficult to provide a sufficiently large N to ensure sufficient $M_{\text{eff}}$ correction. In genetic studies, it has been stated that the $M_{\text{eff}}$ correction can be applied to both single- and multiple-subject analyses and to multivariate analyses, depending on the approach to the correlation matrix.^27^^,^^38^ The effectiveness of the $M_{\text{eff}}$ approach for these statistical tests needs to be verified with fNIRS data. Therefore, this study alone cannot definitively state the effectiveness of the $M_{\text{eff}}$ approach in the face of increasing multichannelization and diversification of experimental designs in fNIRS studies.

Furthermore, the $M_{\text{eff}}$ method, similar to Bonferroni correction, the Holm correction, and the FDR method, cannot distinguish between functional brain activity and physiological interference. fNIRS signals contain physiological interference from sources, such as respiration, heartbeat, and blood pressure, which are unrelated to neurovascular coupling.^39^^–^^41^ Neglecting physiological interference leads to false positives, where the detection of a hemodynamic response is incorrectly attributed to functional brain activity, or false negatives, where brain activity is masked.^41^ Thus fNIRS signals should be appropriately preprocessed before calculating summary data such as β-values and average values of Hb signals. Although the fNIRS data in this study were preprocessed with Wavelet-MDL,^36^ methods such as short-channel regression, PCA, and ICA have, in recent years, been employed to remove extraneous scalp hemodynamics.^4^^,^^41^ Further studies are required to determine how the $M_{\text{eff}}$ values are affected by these different preprocessing procedures.

Finally, the $M_{\text{eff}}$ method is compatible with parametric tests commonly used to analyze fNIRS data, as it employs Pearson’s correlation coefficient. However, due to uncertainties in the distribution of fNIRS signals and variations in responses between participants, parametric model assumptions may not always be assumed. When applying a nonparametric model, a resampling-based approach, such as permutation and bootstrap tests, or the Max-T correction,^42^ is generally used to control for FWER. In such cases, there is no need to use the $M_{\text{eff}}$ method. On the other hand, the $M_{\text{eff}}$ method may be applicable when conducting nonparametric tests utilizing rank orders, such as the Wilcoxon rank sum test and Mann–Whitney U test, for each channel. In such cases, the use of Spearman’s rank correlation coefficient is more appropriate. Further studies are needed for validation of the $M_{\text{eff}}$ method with these nonparametric tests.

## 5 Conclusion

Multichannelization and increasing diversity in experimental designs of fNIRS studies inevitably lead to FWE correction issues in exploratory analyses. Typically, Bonferroni correction and its derivatives are too stringent: The first round of correction always begins by dividing p by the number of channels. Although $M_{\text{eff}}$ has been introduced to provide moderate solutions for FWE correction issues, it is not sufficient for small sample size studies due to rank deficiency in eigenvalues. We used simulations with a typical exponential model to explore the possibility of predicting $M_{\text{eff}}$ values in regions unaffected by rank deficiency for small N. We concluded that predicted values close to the target values could be obtained in these simulations. This demonstrates the potential applicability of the $M_{\text{eff}}$ correction method for fNIRS data with a small N. Thus the $M_{\text{eff}}$ correction, taking interchannel correlation into consideration, could serve as a promising alternative to Bonferroni correction and its derivatives even for data from small sample sizes.

## Supplementary Material



## Data Availability

The code and sample data needed to conduct this analysis is accessible in our GitHub repository at https://github.com/Dan-brain-Lab
