# Supplementary material for: Adjusting effective multiplicity (Meff) for family-wise error rate in functional near-infrared spectroscopy data with a small sample size
Source: Neurophotonics. 2024 Jul 27;11(3):035004. doi: 10.1117/1.NPh.11.3.035004 (PMC11283272; doi:10.1117/1.NPh.11.3.035004)
Supplement: Supplementary file 1 [file NPh_011_035004_SD001.pdf]

Supplementary Material

Further adjusting effective multiplicity (M_{eff}) for family-wise error rate in fNIRS data with a small sample size

Yuki Yamamoto, Wakana Kawai, Tatsuya Hayashi, Minako Uga, Yasushi Kyutoku, and Ippeita Dan

1 Introduction

In the resampling and predictive simulations conducted in Experiment 2 of the main article, it was suggested that the prediction of valid M_{eff} is feasible when the sample size (N) is more than 60 to 70% of the number of functional near-infrared spectroscopy (fNIRS) channels (M). Specifically, it was observed that the prediction error remained below or around 1 (in multiplicity) or 5% when N ranged from 30 to 40 in datasets with 44 or 52 channels.

However, when N constitutes a low percentage of M, the standard deviation (SD) of the prediction increased considerably. This elevates the risk of either insufficient correction due to underestimation or excessive correction due to overestimation of M_{eff}. In fNIRS analysis, obtaining a sufficient N against M for an accurate prediction can sometimes be difficult. This issue becomes particularly notable in multi-channel measurements utilizing several dozen to over 100 channels. To minimize the

prediction error using the exponential model, an adequate *N* is necessary. However, if achieving this is difficult, the applicability of the M_{eff} correction should also be assessed.

In such cases, a modification through penalties can prevent the risk of insufficient correction by accounting for the underestimation of M_{eff} while accepting the risk of conservative correction. In this supplementary material, we examine the possibility of applying penalties based on the ratio of *N* to *M* through the results of Experiment 2. We will introduce a function, $Penalty = f\,(N/M)$, designed to compute the penalty value from the *N* to *M* ratio.

2 Methods

2.1 Calculation of penalty value

To derive the function, we utilized the *SD* of the ratio between the target M_{eff} value and the predicted M_{eff} value for each *N*. The target value is denoted as V_t, and the predicted value is V_p^i (*i* ranging from 1 to 1000). This ratio ($g^{N,i}$) can be interpreted as an indicator of prediction accuracy.

$$Target\ value = V_t\ , \tag{1}$$

$$predicted\ value = V_p^i\ , \tag{2}$$

$$g^{N,i} = \frac{V_p^i}{V_t}\ (i = 1, 2, \dots 1000), \tag{3}$$

A $g^{N,i}$ greater than 1 indicates an underestimation by the prediction, whereas a value less than 1 suggests an overestimation. Thus, the *SD* of $g^{N,i}$ reflects the variability in prediction accuracy for each *N* of the simulations in Experiment 2.

$$\overline{g^N} = \frac{1}{1000}\sum_{i=1}^{1000} g^{N,i}, \tag{4}$$

$$SD_g^{N,\cdot} = \sqrt{\frac{\sum_{i=1}^{n}\left(\overline{g^N} - g^{N,i}\right)}{n}}, \tag{5}$$

It is important to note that, here, the penalty was calculated by dividing target values by predicted values. In actual experiments, the target values are unknown. In this simulation, however, both target and predicted values could be obtained. The penalty was calculated based on the predicted value.

2.2 Derivation of functions

The $g^{N,i}$ for each *N* was calculated 1000 times using four data sets, which are described in the main article, to establish the function, *Penalty* = *f* (N/M). Subsequently, the $SD_g^{N,\cdot}$ for each *N* was calculated. Penalty values obtained from the four datasets were combined to plot the relationship between *N* and *M*. From the approximate curve, *Penalty* = *f* (*N*/*M*) was derived. The function curve enabled the calculation of penalty values for a specific *M* and *N* within any given dataset.

2.3 Calculation of double-adjusted M_{eff}

The double-adjusted M_{eff} value can be calculated by applying the derived penalty value as follows:

$$Double - adjusted\ M_{\text{eff}} = Adjusted\ M_{\text{eff}} \times (1 + Penalty), \tag{6}$$

$$Penalty = f\left(\frac{N}{M}\right), \tag{7}$$

3 Results

We plotted the penalty values obtained from the four datasets against each *N*/*M* ratio with an exponential approximation applied. The root mean square error (*RMSE*) exhibited a small value at 0.037, below 0.1. This analysis led to the derivation of a formula to calculate the penalty based on the *N* to *M* ratio.

$$Penalty = 2.246e^{-6.229N/M} \quad (8)$$

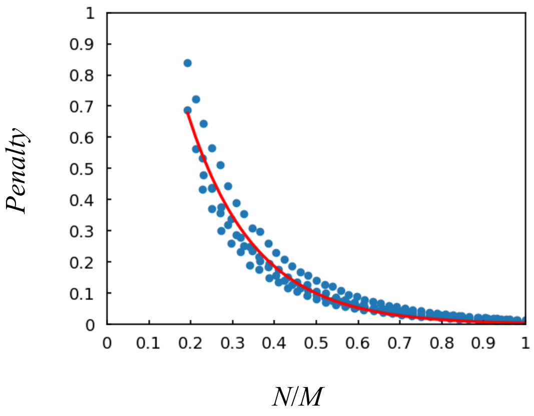

Fig. 1 The blue dots represent penalty values computed across four datasets, and the red line is an exponential approximation.

4 Practical examples

Here, we will describe practical examples of the computation of double-adjusted M_{eff} using Go/No-go task data, which is described in the main text. In the predictive simulations of Experiment 2, the average, minus *SD*, of predicted values represents potentially underestimated predictions in actual analyses. We calculated the double-adjusted M_{eff} assuming that this value (the average, minus *SD*, of

the predicted value for each *N*) could represent predicted values, from datasets with smaller sample sizes (*N* = 10, 15, 20), which were randomly selected from the original sample (*N* = 66). The target value of this data is 24.2 ± 0.53.

Example 1

・For $N = 10$, $M = 44$, the *N*/*M* ratio is 0.23

・Applying the Penalty function $f(0.23) = 2.246^{-6.229 \times 0.23}$, we obtain a penalty value of 0.54

・Underestimated M_{eff} = average – *SD* of predicted M_{eff} = 23.79 - 8.276 = 15.51

・Thus, the double-adjusted M_{eff} is calculated as 15.51 × (1 + 0.54) = 23.89

Example 2

・For $N = 15$, $M = 44$, the *N*/*M* ratio is 0.34

・*Penalty* = $f(0.34) = 2.246^{-6.229 \times 0.34} = 0.27$

・Underestimated M_{eff} = average – *SD* of predicted M_{eff} = 23.94 - 4.66 = 19.28

・Double-adjusted M_{eff} =19.28 × (1 + 0.27) = 24.49

Example 3

・For $N = 20$, $M = 44$, the *N*/*M* ratio is 0.45

・*Penalty* = $f(0.45) = 2.246^{-6.229 \times 0.45} = 0.14$

・Underestimated M_{eff} = average – *SD* of predicted M_{eff} = 23.84 – 2.546 = 21.29

・Double-adjusted M_{eff} =21.29 × (1 + 0.14) = 24.28

5 Discussion

In this supplementary material, we have proposed the modification of predicted M_{eff} values by introducing a penalty value when *N* is below the threshold recommended for accurate prediction. From the simulation results of Experiment 2, a function *penalty* = *f* (*N*/*M*) was derived to calculate the penalty value based on the ratio of *N* to *M*. This modification through penalty values can prevent underestimation of predicted M_{eff} values obtained from a dataset with a small *N*. In the practical examples, underestimated M_{eff} values were adjusted to be closer to the target values. The graph of *penalty* = *f* (*N*/*M*) (Fig. 1) shows that the penalty value drops below 0.1 when *N*/*M* is about 0.5. When *N* exceeds this point, the value added by the penalty approaches the *SD* of target values, suggesting that the need for a penalty value has decreased. On the other hand, the penalty value increased when *N*/*M* ratios were below this point, leading to conservative corrections similar to the Bonferroni method. Therefore, to leverage the advantages of the M_{eff} correction method, it is desirable to obtain as large a *N* as possible.
